# The use of ketamine on emergence agitation in children: a systematic review and meta-analysis

**DOI:** 10.1016/j.bjane.2025.844675

**Published:** 2025-08-28

**Authors:** Ka Ting Ng, Jun Chuen Hui, Wan Yi Teoh, Ina Ismiarti Shariffuddin, Mohd Fitry Zainal Abidin

**Affiliations:** a University of Malaya, Department of Anaesthesiology, Kuala Lumpur, Malaysia; b Queen’s University Belfast, Northern Ireland, United Kingdom

**Keywords:** Emergence agitation, Emergence delirium, Ketamine, Meta-analysis, Pain, Systematic review

## Abstract

**Background:**

Ketamine is believed to reduce the incidence of emergence agitation in children after surgery. However, recent studies reported contradictory findings. Thus, the primary objective of this review and meta-analysis was to investigate the use of ketamine in the reduction of emergence agitation in children undergoing surgery or procedure.

**Methods:**

MEDLINE, EMBASE and CENTRAL were systematically searched from their inception date until March 2024. Randomized controlled trials comparing intravenous ketamine and placebo in children were sought. Observational studies, editorial letters or case reports were excluded.

**Results:**

Seventeen studies (1515 patients) were included. Children who received ketamine were reported to have a significantly lower incidence of emergence agitation (OR = 0.27, 95% Confidence Interval: 0.16 to 0.45, *p* < 0.00001, I^2^ = 61%, certainty of evidence: very low). As compared to placebo, the ketamine group had a significantly lower postoperative pain score (MD = -2.28, 95% Confidence Interval -3.68 to -0.87, *p* = 0.001, I^2^ = 91%, certainty of evidence: very low). However, no significant differences were observed in the incidence of postoperative nausea and vomiting, desaturation, and laryngospasm.

**Conclusion:**

This meta-analysis highlights the potential benefits of ketamine in the reduction of emergence agitation in children undergoing surgery or diagnostic procedures. However, high degrees of heterogeneity and low certainty of evidence limit the recommendations of the routine use of ketamine in the prevention of emergence agitation in children. Further high-quality randomized controlled trials are warranted before routine use can be recommended.

**PROSPERO registration:**

CRD42024523680.

## Introduction

Emergence agitation, also known as emergence delirium, is a temporary state of psychomotor agitation and perceptual disruption that occurs after the emergence from general anesthesia.^1^ First described by Eckenhoff and colleagues in 1961, it presents a significant clinical challenge, particularly in pediatric patients.^2^ Its incidence in the general population varies from 5 % to 30 %, but it could be reported from 20 % to 80 % in the pediatric population.^3^ Though it is often self-limiting and reversible, it poses great risks to healthcare staffs, family members and patients as it could impose harm to patients and surroundings, such as pulling out catheters, drains and intravenous lines, which may disrupt patient care and compromise patient safety.^4^

There are various possible risk factors for emergence agitation in children, mainly patient-related (preschool age, high pre-operation patient/caretaker anxiety level), surgery-related (type of surgery), and anesthesia-related (pain level, lack of premedication, choice of anesthetic).^5^ The management of pain in children undergoing surgery or diagnostic procedures are crucial as studies show patients with moderate and severe postoperative pain often associated with emergence agitation.^6^ One of the most common tools being used to assess the severity of emergence agitation, the Pediatric Anesthesia Emergence Delirium (PAED) scale, allows clinicians to differentiate between pain-related agitation and post-operative delirium based on five behavioral indicators namely eye contact, purposeful actions, awareness of surroundings, restlessness and inconsolability.^7^^,^^8^ Several studies have also demonstrated the positive correlation of lower rate of emergence agitation and satisfactory pain relief.^8^, ^9^, ^10^

Ketamine is a N-Methyl-d-Aspartate (NMDA) receptor antagonist.^11^ One of its enantiomers (S-ketamine) has been one of the main choices as general anesthetic in short procedures due to its wide margin of safety, analgesic, sedative and sympathomimetic effect.^12^ Its use in prevention of emergence agitation has been described in numerous adults, and pediatric studies have demonstrated positive effects of ketamine in the reduction of emergence agitation in children.^12^, ^13^, ^14^. To the best of our knowledge, there has not been an up-to-date and relevant review since 2019, which previously advised caution due to substantial heterogeneity and potential for type I error.^15^ New clinical studies with more robust methodologies have since emerged to provide more clarity into the potential of ketamine in reducing emergence agitation. Thus, they underscore the need for an up-to-date meta-analysis to re-examine and consolidate all the available evidence.

We hypothesized that intravenous ketamine reduces the incidence of emergence agitation in children. Therefore, the primary objective of this meta-analysis was to re-investigate the evidence on use of ketamine in the incidence of emergence agitation in the pediatric population. Secondary objectives included the effect of ketamine on recovery time (defined as time required to reach Aldrete score of ≥ 9), pain score at the arrival of recovery unit, incidence of nausea/vomiting, desaturation, and laryngospasm.

## Material and methods

### Study design

This review was conducted following the Cochrane Handbook for Systematic Reviews of Interventions.^16^ It is reported according to the Preferred Reporting Items for Systematic reviews and Meta-Analyses (PRISMA 2020) reporting guideline.^17^ Our review protocol was registered and published in the PROSPERO database (CRD42024523680).

### Search strategy

Literature search for relevant articles published in CENTRAL, EMBASE and MEDLINE was conducted in March 2024. ClinicalTrials.gov and the WHO International Clinical Trials Registry Platform were thoroughly searched for any ongoing trials. Search strategy for this review is shown in the Online Supplementary Material (named Supplementary Table S1).

### Inclusion and exclusion criteria

Inclusion criteria were: (a) Parallel arm Randomized Controlled Trials (RCTs); (b) Pediatric population less than 18 years old; (c) Comparison between intravenous ketamine and placebo/saline. No language restrictions were applied.

Exclusion criteria for this study were: (a) Non-human studies, observational studies, case reports, case reviews, cross-over RCTs; (b) Adult population age 18 and above; (c) Parent refusal, subject with mental health conditions or developmental delay; (d) Other forms of ketamine being used (oral, intranasal, epidural). Cross-over trials were excluded from our review to minimize potential bias of the pharmacokinetics of ketamine in the cross-over patients.

### Study selection and data extraction

Both authors (JCH, WYT) screened titles and abstracts against the eligibility criteria for this meta-analysis with the Mendeley Reference Software. Full text articles which fulfilled the criteria were obtained for further screening by two authors (JCH, WYT). Any discrepancies were then resolved by the third author (KTN). Data extraction was then performed by two authors (JCH and WYT) independently using a standardized online data extraction form which was designed by the third author (KTN). The following data were extracted: Author name, publication year, study design, country, sample size, mean population age, clinical setting and the dosage of ketamine used.

### Quality assessment

The Risk of Bias (RoB) assessment tool is developed by the Cochrane to assess the risk of bias for randomized controlled trials based on five domains, namely randomization process; any deviations from the intended interventions; any missing outcome data; the measurement of the outcome and the selection of the reported result.^18^ The RoB1 tool was utilized in this review by two authors (JCH and WYT) independently, with a third author (KTN) consulted to resolve any conflicts.

### Measured outcomes

The primary outcome for this review was the incidence of emergence agitation in pediatric patients. In studies where the Pediatric Anesthesia Emergence Delirium (PAED) score of ≥ 10, ≥ 12 and ≥ 15 was available, a PAED score of ≥ 10 was used to determine the incidence of emergence agitation due to its high diagnostic sensitivity and specificity.^19^, ^20^, ^21^, ^22^ Other secondary outcomes were recovery time (defined as time required to reach Aldrete score of ≥ 9), pain score (the Children's Hospital of Eastern Ontario Pain [CHEOP] or modified Children's Hospital of Eastern Ontario Pain [mCHEOP] tools) at PACU arrival, incidence of nausea/vomiting, desaturation and laryngospasm.

### Data analysis

Review Manager version 5.3 was used for data pooling in order to generate forest plots.^23^ A p-value of less than 0.05 (two-tail) indicated that the test result was statistically significant. All the reported findings were described as Mean Difference (MD) and Odds Ratio (OR) with 95 % Confidence Interval (95 % CI) for continuous and binary outcomes respectively. The degree of heterogeneity in all measured outcomes was assessed with the I-square (I^2^) test. I^2^-values of < 40 %, 40 %‒60 %, and > 60 % indicated low, moderate and high heterogeneity respectively. If a high heterogeneity degree were present, a random-effect model would be used for data analysis. Otherwise, a fixed-effect model would be applied to all the measured outcomes. As there were different scoring systems on the severity of emergence agitation, a subgroup analysis was performed on the primary outcome of this study.

The GRADE approach was used to assess the quality of evidence for each outcome of the meta-analysis, with the aid of GRADEpro GDT.^24^ The quality of evidence was assessed based on five domains: risk of bias, inconsistency of results, indirectness of evidence, imprecision, publication bias.^25^ Any uncertainty was resolved by third author (KN).

## Results

**Figure 1:**
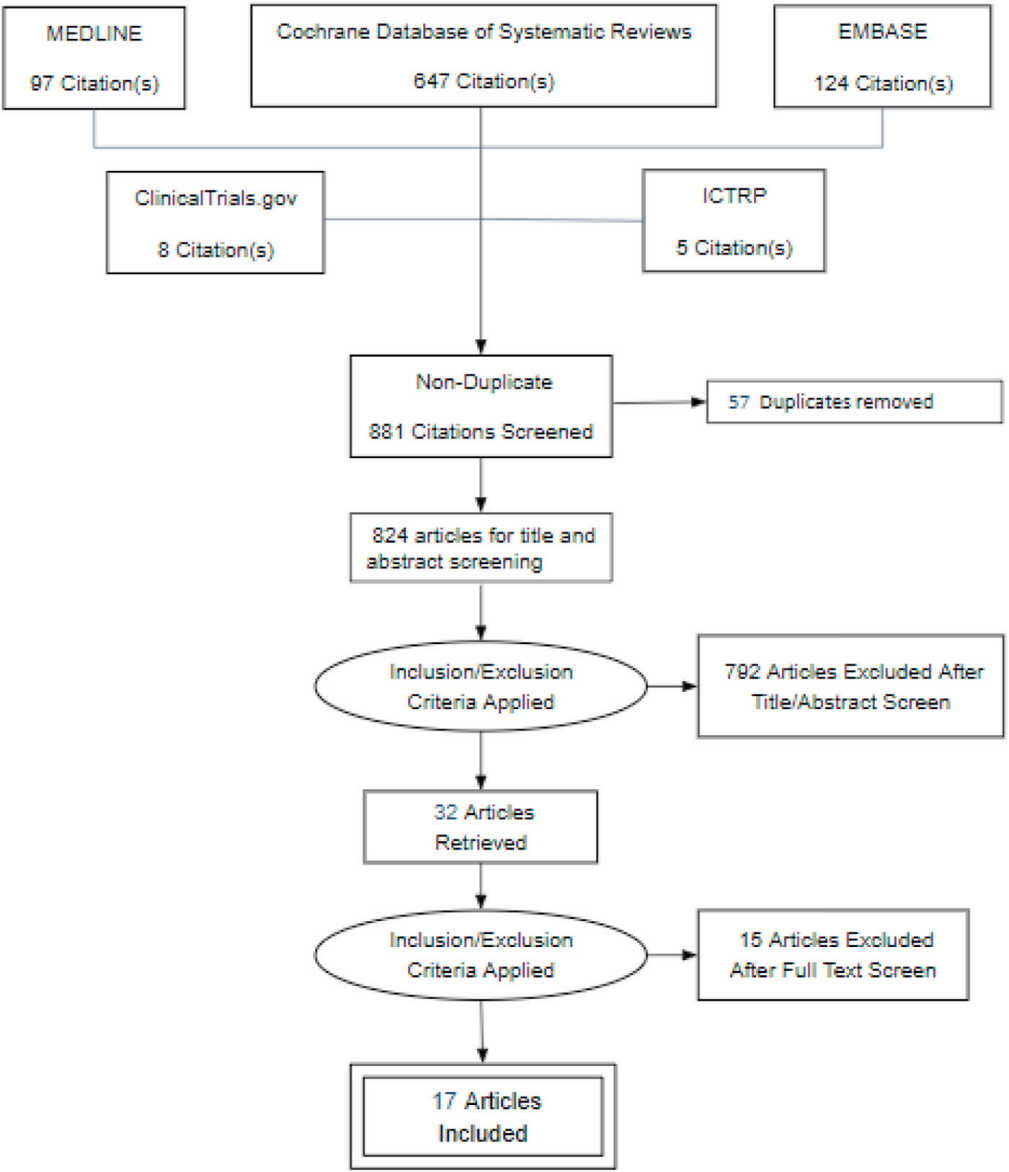
PRISMA flow diagram.

The PRISMA flow diagram illustrates the process of study selection and literature search (Figure 1). A total of 824 articles were retrieved for the title and abstract screening. Among all, thirty-two articles were selected for full text screening. Fifteen studies were excluded from the review (Supplementary Table S2). Seventeen articles with a total of 1515 patients were included in this review. Notable to mention that one relevant ongoing clinical trial was identified during the literature search (TCTR20221024001), which was scheduled to be completed by June 1, 2025 (Supplementary Table S3).

The clinical characteristics of all the included studies are outlined in Table 1. All the 17 studies are single-centered RCTs. Fourteen of these trials were conducted in operating theatres,^9^^,^^10^^,^^12^^,^^14^^,^^26^, ^27^, ^28^, ^29^, ^30^, ^31^, ^32^, ^33^, ^34^, ^35^ whereas the other three studies were in imaging scan rooms.^36^, ^37^, ^38^ In terms of comparators, the majority used ketamine or s-ketamine as comparators, with the exception of three studies that compared patients receiving ketamine-propofol versus propofol only.^30^^,^^32^^,^^38^ Most of the studies administered intravenous ketamine in bolus injection, whereas only two studies gave it bolus followed by infusion.^9^^,^^32^ The dosage used across all the included studies varied from 0.20 mg.kg^-1^ to 1.0 mg.kg^-1^. The main choice of general anesthesia was sevoflurane in the 14 studies.^9^^,^^12^^,^^14^^,^^27^, ^28^, ^29^, ^30^, ^31^^,^^33^, ^34^, ^35^, ^36^, ^37^, ^38^ In terms of the emergence agitation assessment tools, the PAED score was used in seven of the studies.^9^^,^^12^^,^^28^^,^^31^^,^^32^^,^^37^^,^^38^ Emergence Agitation Score (EAS) in three studies,^29^^,^^33^^,^^36^ Aono’s Four-point Scale in six studies,^10^^,^^14^^,^^26^^,^^27^^,^^30^^,^^34^ and Richmond Agitation-Sedation Scale (RASS) in one study.^35^ The overview of data analysis of primary and secondary outcomes is outlined in Table 2. Summary of findings and certainty of evidence using GradePRO is illustrated in Table 3.

**Table 1:** Clinical characteristics of included studies.

Author	Year	Design	Age (Mean ± *S*.D.) [Year]	Comparator	Ketamine Dose	Regime of ketamine	Timing of ketamine	Control	Choice of Anesthesia	Type of EA scale	% of EA (ketamine/ placebo)	Type of procedure	Setting	Country	n
Dalens^36^	2006	Single-center RCT	K: 36.8 ± 17.9 mo; C: 29.1 ± 20.2 mo	Ketamine	0.25 mg.kg^-1^	Bolus	At the end of procedure	Saline	Sevoflurane	Emergence agitation scale ≥ 4	0 % / 10.7 %	Elective MRI	MRI room	Canada	61
Abu-Shahwan^12^	2007	Single-center RCT	K: 5.3 ± 0.9; C: 5.4 ± 0.8	Ketamine	0.25 mg.kg^-1^	Bolus	10 min before the end of surgery	Saline	Sevoflurane	PAED ≥ 15	16.6 % / 34.2 %	Dental repair with no extraction	Operation Theatre	Canada	80
Lee^14^	2010	Single-center RCT	K0.25: 5.0 ± 0.4; K0.5: 5.0 ± 0.4; C: 4.8 ± 0	Ketamine	K0.25: 0.25 mg.kg^-1^; K0.5: 0.5 mg.kg^-1^	Bolus	Before the end of the surgery	Saline	Sevoflurane	Aono's four-point scale > 2	20 % / 80 %	Adenotonsillectomy	Operation Theatre	Korea	90
Jeong^26^	2012	Single-center RCT	K1.0: 5.0 ± 0.4; K0.5: 5.0 ± 0.4; C: 4.8 ± 0.4	Ketamine	K0.5: 0.5 mg.kg^-1^; K1.0: 1.0 mg.kg^-1^	Bolus	Before entering OT; 10 min before completion of surgery	Saline	Desflurane	Aono's four-point scale > 2	25 % / 85 %	Ophthalmic surgery	Operation Theatre	Korea	60
Abdelhalim^27^	2013	Single-center RCT	K:5.1 ± 1.6; C: 4.8 ± 1.9	Ketamine	0.5 mg.kg^-1^	Bolus	10 min before the end of surgery	Saline	Sevoflurane	Aono's four-point scale > 2	15 % / 42.5 %	Tonsillectomy ± Adenoidectomy	Operation Theatre	Saudi Arabia	80
Chen^9^	2013	Single-center RCT	K: 4.2 ± 1.2; C: 4.3 ± 1.1	Ketamine	1.0 mg.kg^-1^	Bolus + infusion	After induction	Saline	Sevoflurane	PAED ≥ 10	29.6 % / 70.8 %	Strabismus surgery	Operation Theatre	China	51
Eghbal^10^	2013	Single-center RCT	K: 9.1 ± 3.3; C: 8.2 ± 3.1	Ketamine	0.25 mg.kg^-1^	Bolus	Before the end of the surgery	Saline	Isoflurane	Aono's four-point scale > 2	30.3 % / 90.9 %	Adenotonsillectomy	Operation Theatre	Iran	66
Ozcan^28^	2014	Single-center RCT	K: 4.0 ± 1.6; C: 4.8 ± 3	Ketamine	0.25 mg.kg^-1^	Bolus	10 min before the end of surgery	Saline	Sevoflurane	PAED ≥ 10	30 % / 55 %	Inguinal hernia repair, circumcision or orchidopexy	Operation Theatre	Turkey	40
Rashad^29^	2014	Single-center RCT	K: 26.9 ± 9.1 mo; C: 24.6 ± 10.2 mo	Ketamine	0.25 mg.kg^-1^	Bolus	Before the end of the surgery	Saline	Sevoflurane	Emergence agitation scale ≥ 4	20 % / 40 %	Hypospadias repair	Operation Theatre	Egypt	40
Rizk^30^	2014	Single-center RCT	K: 4.3 ± 1.5; C: 4.1 ± 1.3	Ketamine - Propofol	0.25 mg.kg^-1^	Bolus	Before the end of the surgery	Usual treatment (propofol)	Sevoflurane / Propofol	Aono's four-point scale > 2	16.7 % / 23.3 %	Tonsillectomy ± Adenoidectomy	Operation Theatre	Egypt	60
Moawad^37^	2015	Single-center RCT	K0.25: 4.3 ± 1.4; K1.0: 4.2 ± 1.5; C: 4.6 ± 1.4	Ketamine	K0.25: 0.25 mg.kg^-1^; K1.0: 1.0 mg.kg^-1^	Bolus	before induction; 10 min prior to the end of procedure	Saline	Sevoflurane	PAED ≥ 10	–	Elective MRI	MRI room	Egypt	120
Ozturk^31^	2016	Single-center RCT	K: 4.3 ± 1.3; C: 4.0 ± 1.6	Ketamine	0.5 mg.kg^-1^	Bolus	Post-extubation	Saline	Sevoflurane	PAED ≥ 15	‒	Fiberoptic bronchoscopy	Operation Theatre	Turkey	46
Schmitz^38^	2018	Single-center RCT	K: 4.1 ± 0.8; C: 3.7 ± 0.8	Ketamine - Propofol	1.0 mg.kg^-1^	Bolus	Before the procedure	Usual treatment (propofol)	Sevoflurane / Propofol	PAED ≥ 10	4.3 % / 1.8 %	Elective MRI	MRI room	Switzerland	331
Jalili^32^	2019	Single-center RCT	K: 7.3 ± 2.1; P: 7 ± 2.1	Ketamine - Propofol	25 mg.kg^-1^.min^-1^	Infusion	During operation (maintenance of anesthesia)	Usual treatment (propofol)	Sodium thiopental + Atracurium	PAED ≥ 12	20.9 % / 27.3 %	Tonsillectomy	Operation Theatre	Iran	87
Ibrahim^33^	2023	Single-center RCT	K: 5.6 ± 1.32; C: 5.7 ± 1.3	Ketamine	0.25 mg.kg^-1^	Bolus	Post-extubation	Saline	Sevoflurane	5 step EA scale > 5 for > 3 mins	10 % / 16.6 %	Tonsillectomy ± Adenoidectomy	Operation Theatre	Egypt	90
Chen^34^	2023	Single-center RCT	K: 5.0 ± 1.4; C: 5.4 ± 1.4	S-ketamine	0.2 mg.kg^-1^	Bolus	Upon completion of surgery	Saline	Sevoflurane	Aono’s four-point scale ≥ 3	7.4 % / 22.2 %	Tonsillectomy ± Adenoidectomy	Operation Theatre	China	108
Qiu^35^	2023	Single-center RCT	K: 35.6 ± 29.3 mo; C: 40.3 ± 35.8 mo	S-ketamine	0.5 mg.kg^-1^	Bolus	During induction	Saline	Sevoflurane	RASS > 0 [EA]	45.3 % / 78.9 %	Laparoscopic hernia repair	Operation Theatre	China	105

**Table 2:** Meta-analytic findings of primary and secondary outcomes.

	Outcomes	Trials	n	I^2^ ( %)	MD/OR (95 % CI)	p
**1**	Incidence rate of emergence agitation	15	1319	61	0.27 [0.16, 0.45]	< 0.00001
**1.1**	Subgroup analysis by three different scoring systems					
	PAED	5	589	56	0.51 [0.23, 1.13]	0.10
	Aono’s four-point scale	6	464	64	0.14 [0.06, 0.33]	< 0.00001
	Emergence agitation scale	4	266	0	0.28 [0.15, 0.54]	0.0001
**2**	Pain score upon arrival of PACU	6	429	94	−2.28 [−3.68, −0.87]	0.001
**3**	Recovery time (time required to reach Aldrete Score of ≥ 9)	12	1108	91	−0.77 [−2.76, 1.21]	0.44
**4**	Nausea/Vomiting	12	1201	0	1.20 [0.81, 1.75]	0.36
**5**	Desaturation	7	817	0	0.95 [0.58, 1.56]	0.84
**6**	Laryngospasm	4	267	0	0.82 [0.24, 2.75]	0.75

PAED, Pediatric Anesthesia Emergence Delirium; PACU, Post-Anesthesia Care Unit; n, Sample size; MD, Mean Difference; OR, Odds Ratio; CI, Confidence Interval; p, p-value.

**Table 3:** Summary of findings table.

Certainty assessment							N° of patients		Effect		Certainty	Importance
N° of studies	Study design	Risk of bias	Inconsistency	Indirectness	Imprecision	Other considerations	Ketamine	Placebo	Relative (95 % CI)	Absolute (95 % CI)		
**Incidence of emergence agitation**												
15	Randomized trials	Very serious^a^	Serious^b^	Serious^c^	Not serious	Dose response gradient	115/689 (16.7 %)	220/630 (34.9 %)	**OR 0.27** (0.16 to 0.45)	**223 fewer per 1000** (from 270 fewer to 155 fewer)	⨁○○○ Very low^a^^,^^b^^,^^c^	
**PAED score at 5min postoperatively**												
4	Randomized trials	Serious^d^	Not serious	Serious^c^	Not serious	Dose response gradient	112	105	–	MD **3.99 lower** (5.03 lower to 2.95 lower)	⨁⨁⨁○ Moderate^c^^,^^d^	
**Pain score at PACU**												
6	Randomized trials	Serious^d^	Very serious^e^	Serious^c^	Not serious	Publication bias strongly suspected dose response gradient^f^	243	186	–	MD **2.28 lower** (3.68 lower to 0.87 lower)	⨁○○○ Very low^c^^,^^d^^,^^e^^,^^f^	
**Discharge time (time to Aldrete score ≥ 9)**												
12	Randomized trials	Serious^d^	Very serious^e^	Not serious	Not serious	Publication bias strongly suspected dose response gradient^f^	566	542	–	MD **0.77 lower** (2.76 lower to 1.21 higher)	⨁○○○ Very low^d^^,^^e^^,^^f^	
**Incidence of nausea/vomiting**												
12	Randomized trials	Serious^d^	Not serious^e^	Not serious	Not serious	Publication bias strongly suspected dose response gradient^f^	69/649 (10.6 %)	50/552 (9.1 %)	**OR 1.20** (0.81 to 1.75)	**16 more per 1000** (from 16 fewer to 58 more)	⨁⨁⨁○ Moderate^d^^,^^e^^,^^f^	
**Incidence of desaturation**												
7	Randomized trials	Not serious	Not serious	Not serious^c^	Not serious	Publication bias strongly suspected dose response gradient^f^	36/406 (8.9 %)	40/411 (9.7 %)	**OR 0.95** (0.58 to 1.56)	**4 fewer per 1000** (from 38 fewer to 47 more)	⨁⨁⨁⨁ High^c^^,^^f^	
**Incidence of laryngospasm**												
4	Randomized trials	Very serious^a^	Not serious	Serious^c^	Not serious	Publication bias strongly suspected dose response gradient^f^	5/135 (3.7 %)	6/132 (4.5 %)	**OR 0.82** (0.24 to 2.75)	**8 fewer per 1000** (from 34 fewer to 70 more)	⨁○○○ Very low^a^^,^^c^^,^^f^	

CI, Confidence Interval; MD, Mean Difference; OR, Odds Ratio.

Explanations.

a The majority of included trials were high risk/unclear risk of bias.

b Heterogeneity > 50 %.

c The sample size of each group was < 300.

d Half of studies were unclear risk of bias.

e Heterogeneity > 80 %.

f Funnel plot showed asymmetrical graphically.

The summary of risk of bias assessment using the RoB1 tool was illustrated in Online Supplementary Table S4. Of the overall risk of bias, seven out of 17 studies displayed low risk,^9^^,^^12^^,^^27^^,^^34^^,^^35^^,^^37^^,^^38^ while the remaining ten studies were deemed unclear.^10^^,^^14^^,^^26^^,^^28^, ^29^, ^30^, ^31^, ^32^, ^33^^,^^36^ Both authors completed this review in accordance with the PRISMA checklist (Supplementary Table S5).

### Primary outcome: incidence of emergence agitation

**Figure 2:**
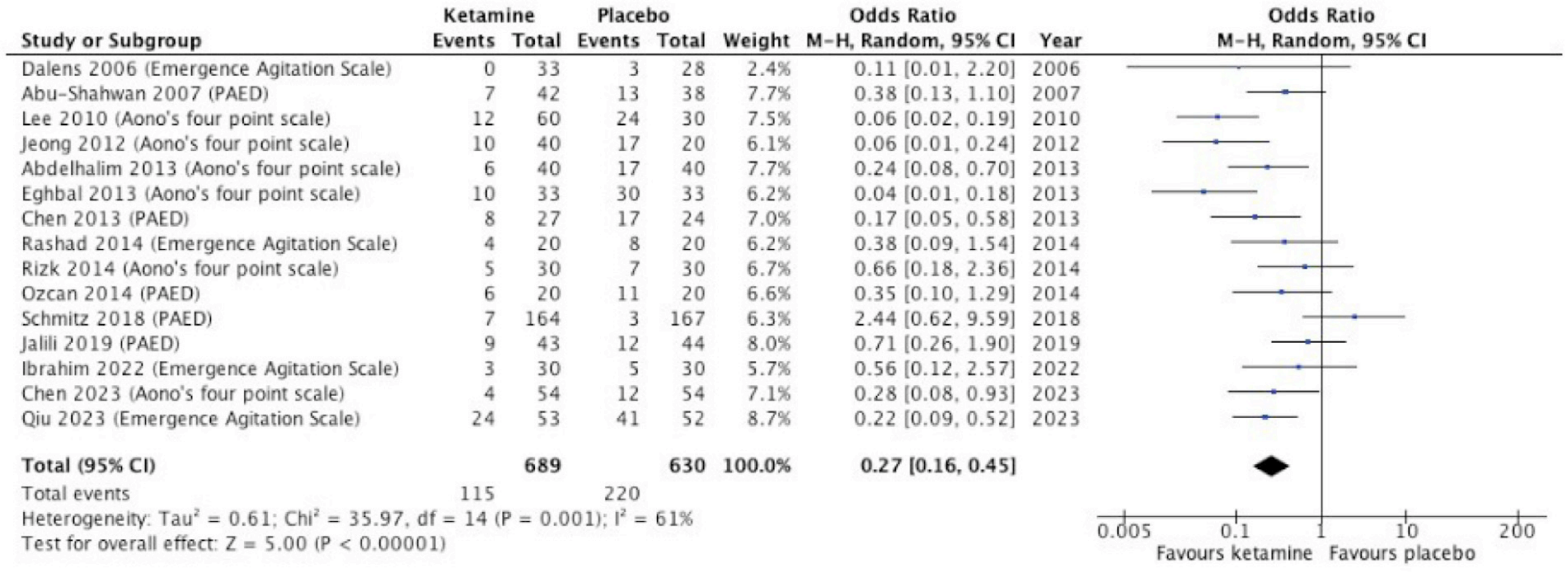
Incidence rate of emergence agitation.

By summarizing the data of 15 studies (*n* = 1319), the incidence of emergence agitation in pediatric patients was 16.7 % in the ketamine group and 34.9 % in the control group. Children who received intravenous ketamine experienced a lower incidence of emergence agitation, with an OR of 0.27 (*p* < 0.00001, 95 % CI 0.16 to 0.45, I^2^ = 61 %) (Figure 2). The certainty of evidence was deemed to be very low due to considerable risk of bias, result inconsistency and imprecision. This finding should be interpreted with great caution given the high substantiality, which might be due to differences in patient age and doses of intravenous ketamine applied across studies.

Subgroup analysis of three main scoring systems for emergence agitation (PAED score, Aono’s four-point scale, EAS) demonstrated a similar result of ketamine’s role in reducing emergence agitation (pooled OR = 0.27, 95 % CI 0.16 to 0.45, *p* < 0.00001) with significant heterogeneity (61 %) (eFigure 1). Chi-Squared test for subgroup differences produced a p-value of 0.09, which indicated no statistically significant differences in results between these scoring systems (I^2^ = 57.8 %). Among the three measuring tools, Aono’s four-point scale has the highest sensitivity as it yielded the most pronounced effect of ketamine in reduction of emergence agitation (pooled OR = 0.14, 95 % CI 0.06 to 0.33, *p* = 0.02, I^2^ = 64 %). The funnel plot did not show evidence of publication bias graphically.

### Secondary outcomes: postoperative pain, recovery time, nausea/vomiting, desaturation, laryngospasm

**Figure 3:**
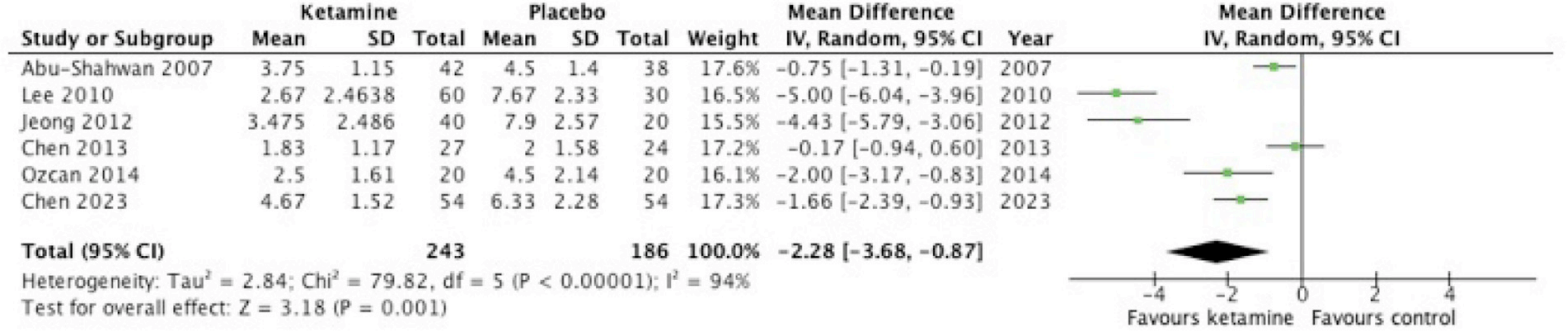
Pain score upon arrival of PACU.

Six studies have examined the effect of intravenous ketamine on postoperative pain in pediatric patients. The pain score upon arrival at the PACU in the ketamine group was significantly lower than in the control group (*n* = 429, *p* = 0.001, MD = −2.28, 95 % CI −3.68 to −0.87) (Figure 3). High degree of heterogeneity was found with an I^2^-value of 94 %. Sensitivity analysis was then performed by removing studies with high or unclear risk of bias, which showed the significance of pain reduction with the intravenous ketamine group (studies = 3, *n* = 239, *p* = 0.03, MD = −0.86, 95 % CI 1.65 to −0.08, I^2^ = 74 %) (eFigure 2). Ketamine did not reduce the duration of recovery time (studies = 12, *n* = 1108, *p* = 0.44, MD = −0.77, 95 % CI −2.76 to 1.21, I^2^ = 91 %) (eFigure 3), although this should also be interpreted with caution due to high heterogeneity.

The pooled result of 12 studies (*n* = 1201) did not find any significant effect of postoperative nausea and vomiting in both the ketamine and control group (*p* = 0.36, OR = 1.20, 95 % CI 0.81 to 1.75) (eFigure 4). No significant effects were observed in the incidence of desaturation (studies = 7, *n* = 817, OR = 0.95, 95 % CI 0.58 to 1.56, *p* = 0.84) (eFigure 5) and laryngospasm (studies = 4, *n* = 267, OR = 0.82, 95 % CI 0.24 to 2.75, *p* = 0.75) (eFigure 6), with low degree of heterogeneity across the three measured outcomes (I^2^ = 0 %). These findings demonstrate that intravenous ketamine in these populations did not result in significant adverse effects, such as desaturation or laryngospasm.

## Discussion

This meta-analysis demonstrated the potential of intravenous ketamine in minimizing the occurrence of emergence agitation and severity of pain following procedures in the pediatric group, although there is considerable degree of heterogeneity and low certainty of evidence in the GRADE framework. Though it did not shorten the duration of recovery, the short-term use of ketamine in the study group also demonstrated a favorable safety profile among children in the reduction of emergence agitation. Clinicians should interpret these results with caution, recognizing that the low certainty of evidence indicates a need for further trials with more robust methodologies to confirm the findings. Until more evidence is available, clinicians should incorporate these findings as part of a broader, evidence-based decision-making process rather than as a definitive recommendation for routine use.

Ketamine’s non-competitive NMDA receptor antagonism reduces excitatory neurotransmission and prevents hyperexcitability after surgery.^39^ It also provides analgesia by acting on opioid receptors and HCN channels.^40^ This review highlights ketamine’s dual role as both an anesthetic and analgesic, which is consistent with several studies in similar settings.^41^, ^42^, ^43^ However, given the considerable heterogeneity across the evidence, clinicians should interpret these results with caution. Our review also emphasizes the need to address study-level sources of heterogeneity that affect the interpretation of ketamine’s clinical utility. While most studies utilized ketamine as monotherapy, there were two recent studies that chose S-ketamine,^34^^,^^35^ and three studies with ketamine-propofol.^30^^,^^32^^,^^38^ S-ketamine, the more potent enantiomer, has higher affinity for NMDA receptors and offers enhanced analgesic and sedation effects. Meanwhile, ketamine-propofol combinations provide more balanced sedation and pain relief as compared to ketamine alone, with propofol contributing antiemetic and sedative profiles, which may reduce the incidence of emergence agitation. These differences in formulation further introduce variability in efficacy and safety outcomes, complicating direct comparisons across studies.

Diagnostic and surgical procedures, ranging from minor diagnostic interventions to major surgeries may have a varied degree of pain and agitation potentials. This variability likely influences the baseline risk of emergence agitation and analgesic requirements, making direct comparisons challenging. While the majority of the studies utilized sevoflurane as maintenance agent, two studies used desflurane^26^ and isoflurane.^10^ The differences in anesthetic agents may contribute to heterogeneity in the incidence of emergence agitation due to their distinct pharmacological profiles.

All included studies used different tools to measure emergence agitation, mainly PAED score, the 5-step EAS, and Aono’s four-point score. This variation across studies reflects the lack of universal agreement on the most appropriate or sensitive tool for evaluating emergence agitation. To address the divergence in findings, we conducted a subgroup analysis to examine whether ketamine’s efficacy remains consistent across different assessment tools. This approach ensures the robustness of evidence and demonstrates the generalizability of ketamine’s effect across varied clinical practices. By accounting for these methodological differences, the analysis helps contextualize our results within the broader clinical landscape, strengthening the recommendations of this review, and providing suggestions for future research and standardized assessment. The subgroup analysis has shown that the effect of ketamine in the reduction of emergence agitation among children was consistent across the three assessment tools, suggesting robustness across differing measurement methods. Our review found that ketamine did not significantly shorten the duration of recovery, with all studies universally agreeing on discharging patients only after an Aldrete score of at least 9 was reached. However, other variabilities, such as differences in patient population, procedural complexities, the use of other adjunct medications, may have prolonged sedation or recovery time, which contributed to inconsistencies in the result.

All trials included in this review administered intravenous ketamine with doses ranging between 0.20 mg.kg^-1^ and 1.0 mg.kg^-1^ before the end of the procedure, which were proven to be adequate for pain control and prevention of emergence agitation without experiencing any noticeable adverse event. Other studies have also acknowledged that a subanesthetic dose between 0.15 and 0.25 mg.kg^-1^ could achieve sufficient analgesic control.^44^, ^45^, ^46^, ^47^ While higher doses in some animal studies (as high as 30 mg.kg^-1^ per day in rats) or chronic exposure of ketamine have raised concerns about ketamine’s potential neurotoxicity,^48^ no research data in a human study has yet conclusively demonstrated any potential clinical risk of a single low dose administration of ketamine in children.^49^ The safety profile is further reinforced by the low incidence of adverse events, such as nausea, vomiting, desaturation and laryngospasm, in the included trials. However, the broad range of study groups (3-months to 15-years) may have contributed to variability in the findings, as younger children metabolize ketamine more rapidly compared to older children,^50^ potentially requiring different dosing regimens.

While ketamine has shown potential in reducing emergence agitation, other anesthetic agents have also been reported to have similar effects in clinical practice, such as midazolam and dexmedetomidine. As an imidazole benzodiazepine, midazolam is widely used for preoperative sedation and lowering anxiety level,^51^ which contributes to lower risk of emergence agitation.^52^ However, it does not possess a significant analgesic property,^53^ making it a less suitable candidate for postoperative pain control. Dexmedetomidine, a selective alpha 2-adrenergic receptor agonist,^54^ displayed both sedative and pain relief characteristics with fewer neurocognitive concerns compared to ketamine.^55^ However, two meta-analyses on dexmedetomidine have revealed that the treatment group significantly prolonged time to extubation, eye-opening, and discharge from the recovery room.^56^^,^^57^ Regardless, further large-scale studies and meta-analyses are warranted to compare these agents and establish whether ketamine’s unique analgesic and sedation abilities hold a clinical advantage against other anesthetic medications in the pediatric population.

Several limitations must be acknowledged in this review: 1) Inconsistencies in variables such as age of subjects, dose of intravenous ketamine used, choice of anesthetic, and scoring tools were used to measure the degree of emergence agitation. 2) The inclusion of smaller sample size clinical trials, which may amplify the effects of intervention and cause false positive findings. 3) We did not evaluate time to extubation or PACU discharge as independent outcomes. 4) Other patient-related risk factors of emergence agitation, such as the preoperative anxiety level of patients and guardians, may have influenced the study findings but were not consistently measured across the included studies. 5) Lack of long-term follow-up data to evaluate potential neurocognitive risks and provide clarity on safety of ketamine use in pediatric populations.

## Conclusions

This systematic review and meta-analysis suggests that intravenous ketamine reduces the incidence of emergence agitation and postoperative pain in children undergoing surgery or diagnostic procedures. However, due to considerable heterogeneity and overall low certainty of evidence, further high-quality randomized controlled trials are required before routine use can be recommended.

## Declaration of competing interest

The authors declare no conflicts of interest.

## Appendix Supplementary materials

Image, application 1

## Footnotes

Supplementary material associated with this article can be found in the online version at doi:10.1016/j.bjane.2025.844675.
