# Supplementary material for: The use of ketamine on emergence agitation in children: a systematic review and meta-analysis
Source: Braz J Anesthesiol. 2025 Aug 28;75(6):844675. doi: 10.1016/j.bjane.2025.844675 (PMC12508892; doi:10.1016/j.bjane.2025.844675)

**BJAN-D-25-00042_ Supplementary Material**

**Supplementary Table S1** Search strategy.

| **EMBASE [Inception ‒ 8^th^ March 2024]** | |
| --- | --- |
| 1 | ketamine.mp. Or exp ketamine/ |
| 2 | delirium.mp. Or exp delirium/or exp postoperative delirium/or exp Emergence delirium/ or emergency agitation.mp. |
| 3 | Exp “clinical trial(topic)”/or exp clinical trial/ or exp “controlled clinical trial(topic)” or trial.mp. Or exp “randomized controlled trial(topic)”/or exp controlled clinical trial/ or exp randomized controlled trial/ |
| 4 | 1 and 2 and 3 |
| 5 | Limit 4 to human |
| 6 | Limit 5 to “remove medline records” |
| **MEDLINE [Inception ‒ 8^th^ March 2024]** | |
| 1 | ketamine.mp. Or exp Ketamine/ |
| 2 | Exp Emergence Delirium/or delirium.mp. Or exp Delirium/ |
| 3 | Emergence agitation.mp. Or exp Emergence Delirium/ |
| 4 | Postoperative agitation.mp. |
| 5 | Exp Randomized Controlled trial/or exp Clinical Trial/or trial.mp. |
| 6 | 2 or 3 or 4 |
| 7 | 1 and 5 and 6 |
| 8 | Limit 7 to humans |
| **CENTRAL [Inception ‒ 8th March 2024]** | |
| #1 | Delirium OR agitation |
| #2 | Ketamine |
| #3 | #1 AND #2 |

**Supplementary Table S2** Clinical characteristics of excluded studies.

| **Author** | **Year** | **Design** | **Reason for exclusion** | **Type of population** | **Country** | **n** |
| --- | --- | --- | --- | --- | --- | --- |
| Ki Young Lee | 1996 | RCT | Wrong route (oral) | Children | Korea | 60 |
| Lee | 2000 | RCT | Wrong route (caudal) | Children | Hong Kong | 32 |
| Weber | 2003 | RCT | Wrong route (caudal) | Children | Germany | 30 |
| Akin | 2005 | RCT | Wrong outcome | Children | Turkey | 60 |
| Da Conceicao | 2006 | RCT | Wrong outcome | Children | Brazil | 90 |
| Shaban | 2008 | RCT | Wrong route (oral) | Children | Egypt | 90 |
| Sinha | 2012 | RCT | Wrong route (caudal) | Children | India | 150 |
| Bilgen | 2014 | RCT | Wrong route (Intranasal) | Children | Turkey | 78 |
| Hayes | 2018 | RCT | Wrong study design | Children | Canada | 65 |
| Abitağaoğlu | 2021 | RCT | Wrong Population | Adult | Turkey | 102 |
| Ustun | 2021 | RCT | Wrong comparison | Children | Turkey | 87 |
| Thakur | 2022 | RCT | Wrong comparison | Children | India | 70 |
| Sahoo | 2022 | RCT | Wrong comparison | Children | India | 141 |
| Wu | 2023 | RCT | Wrong comparison | Children | China | 61 |
| Tang | 2023 | RCT | Wrong comparison | Children | China | 105 |

**Supplementary Table S3** Characteristics of ongoing studies.

| **Author** | **Location** | **Status** | **Recruitment start date** | **Estimated completion date** | **Title** | **Comparator** | **Sample Size** | **Clinical trial number** |
| --- | --- | --- | --- | --- | --- | --- | --- | --- |
| Dr Busapun Sermdumrongsak | Thailand | Not yet recruiting | Pending | 01 June 2025 | The efficacy of ketamine in reducing postoperative pain in children undergoing Cleft Palate (CP) surgery | Ketamine vs. placebo | 46 | TCTR20221024001 |

**Supplementary Table S4** Risk of Bias assessment.

| **Study** | **Cochrane Risk of Bias Tool** | | | | | | | **Overall** |
| --- | --- | --- | --- | --- | --- | --- | --- | --- |
|  | **Sequence generation** | **Allocation concealment** | **Blinding of participants and personnel** | **Blinding of outcome assessment** | **Incomplete outcome data** | **Selective outcome reporting** | **Other sources of bias** |  |
| Dalens 2006 | Unclear | Unclear | Low | Low | Low | Low | Low | Unclear |
| Abu-Shahwan 2007 | Low | Low | Low | Low | Low | Low | Low | Low |
| Lee 2010 | Unclear | Unclear | Low | Low | Low | Low | Low | Unclear |
| Jeong 2012 | Unclear | Unclear | Low | Unclear | Low | Low | Low | Unclear |
| Abdelhalim 2013 | Low | Low | Low | Low | Low | Low | Low | Low |
| Chen 2013 | Low | Low | Low | Low | Low | Low | Low | Low |
| Eghbal 2013 | Unclear | Unclear | Low | Low | Low | Low | Low | Unclear |
| Ozcan 2014 | Unclear | Unclear | Low | Low | Low | Low | Low | Unclear |
| Rashad 2014 | Low | Unclear | Low | Low | Low | Low | Low | Unclear |
| Rizk 2014 | Unclear | Low | Low | Low | Low | Low | Low | Unclear |
| Moawad 2015 | Low | Low | Low | Low | Low | Low | Low | Low |
| Ozturk 2016 | Unclear | Low | Low | Low | Low | Low | Low | Unclear |
| Schmitz 2018 | Low | Low | Low | Low | Low | Low | Low | Low |
| Jalili 2019 | Unclear | Low | Low | Low | Low | Low | Low | Unclear |
| Ibrahim 2022 | Low | Low | Low | Unclear | Low | Low | Low | Unclear |
| Chen 2023 | Low | Low | Low | Low | Low | Low | Low | Low |
| Qiu 2023 | Low | Unclear | Low | Unclear | Low | Low | Low | Low |

**Supplementary Table S5** PRISMA checklist.

| **Section/topic** | **#** | **Checklist item** | **Reported on page #** |
| --- | --- | --- | --- |
| **TITLE** | | |  |
| Title | 1 | Identify the report as a systematic review, meta-analysis, or both. | 1 |
| **ABSTRACT** | | |  |
| Structured summary | 2 | Provide a structured summary including, as applicable: background; objectives; data sources; study eligibility criteria, participants, and interventions; study appraisal and synthesis methods; results; limitations; conclusions and implications of key findings; systematic review registration number. | 2 |
| **INTRODUCTION** | | |  |
| Rationale | 3 | Describe the rationale for the review in the context of what is already known. | 3 |
| Objectives | 4 | Provide an explicit statement of questions being addressed with reference to participants, interventions, comparisons, outcomes, and study design (PICOS). | 3,4 |
| **METHODS** | | |  |
| Protocol and registration | 5 | Indicate if a review protocol exists, if and where it can be accessed (e.g., Web address), and, if available, provide registration information including registration number. | 5 |
| Eligibility criteria | 6 | Specify study characteristics (e.g., PICOS, length of follow-up) and report characteristics (e.g., years considered, language, publication status) used as criteria for eligibility, giving rationale. | 5 |
| Information sources | 7 | Describe all information sources (e.g., databases with dates of coverage, contact with study authors to identify additional studies) in the search and date last searched. | 5 |
| Search | 8 | Present full electronic search strategy for at least one database, including any limits used, such that it could be repeated. | 5 |
| Study selection | 9 | State the process for selecting studies (i.e., screening, eligibility, included in systematic review, and, if applicable, included in the meta-analysis). | 5 |
| Data collection process | 10 | Describe method of data extraction from reports (e.g., piloted forms, independently, in duplicate) and any processes for obtaining and confirming data from investigators. | 6 |
| Data items | 11 | List and define all variables for which data were sought (e.g., PICOS, funding sources) and any assumptions and simplifications made. | 6 |
| Risk of bias in individual studies | 12 | Describe methods used for assessing risk of bias of individual studies (including specification of whether this was done at the study or outcome level), and how this information is to be used in any data synthesis. | 6 |
| Summary measures | 13 | State the principal summary measures (e.g., risk ratio, difference in means). | 6 |
| Synthesis of results | 14 | Describe the methods of handling data and combining results of studies, if done, including measures of consistency (e.g., I^2^) for each meta-analysis. | 6,7 |
| Risk of bias across studies | 15 | Specify any assessment of risk of bias that may affect the cumulative evidence (e.g., publication bias, selective reporting within studies). | 6 |
| Additional analyses | 16 | Describe methods of additional analyses (e.g., sensitivity or subgroup analyses, meta-regression), if done, indicating which were pre-specified. | 6 |
| **RESULTS** | | |  |
| Study selection | 17 | Give numbers of studies screened, assessed for eligibility, and included in the review, with reasons for exclusions at each stage, ideally with a flow diagram. | 8 |
| Study characteristics | 18 | For each study, present characteristics for which data were extracted (e.g., study size, PICOS, follow-up period) and provide the citations. | 8 |
| Risk of bias within studies | 19 | Present data on risk of bias of each study and, if available, any outcome level assessment (see item 12). | 8 |
| Results of individual studies | 20 | For all outcomes considered (benefits or harms), present, for each study: (a) simple summary data for each intervention group (b) effect estimates and confidence intervals, ideally with a forest plot. | 5-6 |
| Synthesis of results | 21 | Present results of each meta-analysis done, including confidence intervals and measures of consistency. | 8,9,10 |
| Risk of bias across studies | 22 | Present results of any assessment of risk of bias across studies (see Item 15). | 8,9 |
| Additional analysis | 23 | Give results of additional analyses, if done (e.g., sensitivity or subgroup analyses, meta-regression [see Item 16]). | 8,9 |
| **DISCUSSION** | | |  |
| Summary of evidence | 24 | Summarize the main findings including the strength of evidence for each main outcome; consider their relevance to key groups (e.g., healthcare providers, users, and policy makers). | 11,12,13 |
| Limitations | 25 | Discuss limitations at study and outcome level (e.g., risk of bias), and at review-level (e.g., incomplete retrieval of identified research, reporting bias). | 13 |
| Conclusions | 26 | Provide a general interpretation of the results in the context of other evidence, and implications for future research. | 13 |
| **FUNDING** | | |  |
| Funding | 27 | Describe sources of funding for the systematic review and other support (e.g., supply of data); role of funders for the systematic review. | 14 |

**eFigure 1** Subgroup analysis of primary outcome.

**
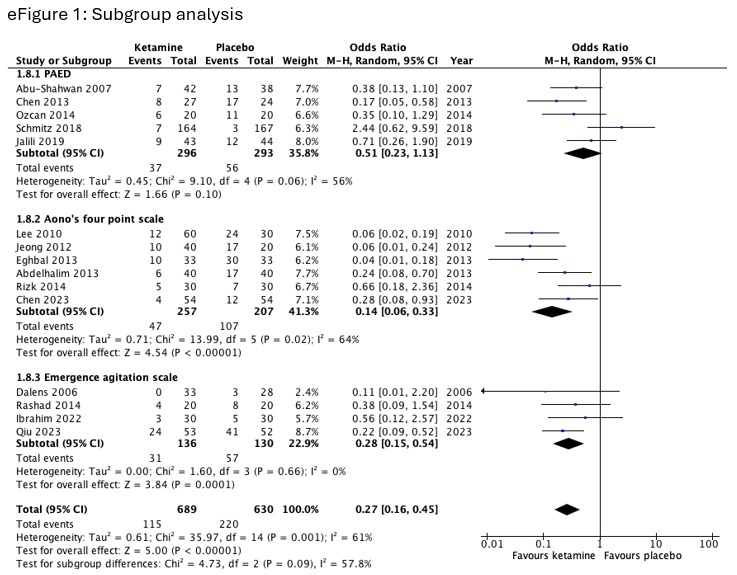
**

**eFigure 2** Sensitivity analysis of postoperative pain score.

**
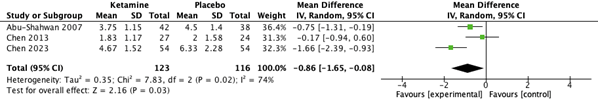
**

**eFigure 3** Recovery time (time required to reach Aldrete score of ≥9).


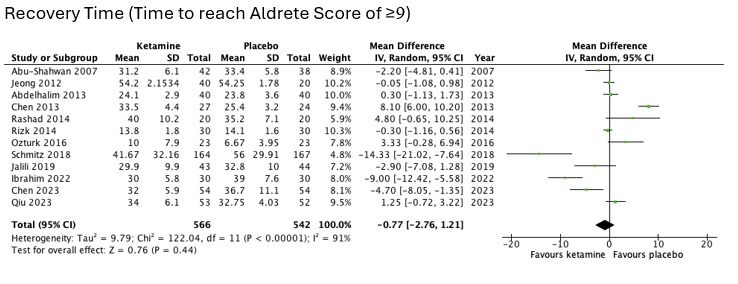


**eFigure 4** Nausea and vomiting.


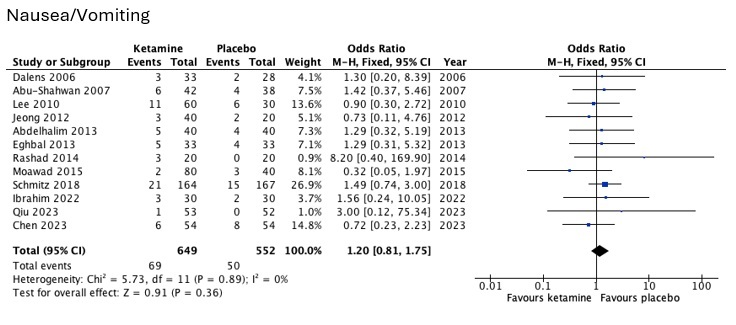


**eFigure 5** Desaturation.


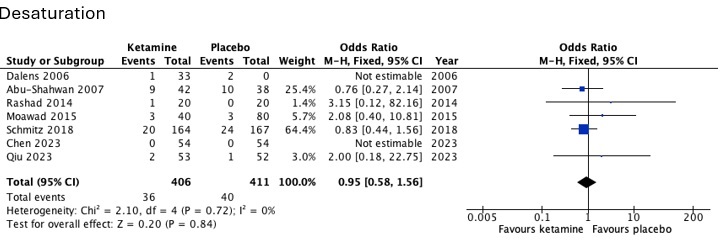


**eFigure 6** Laryngospasm.


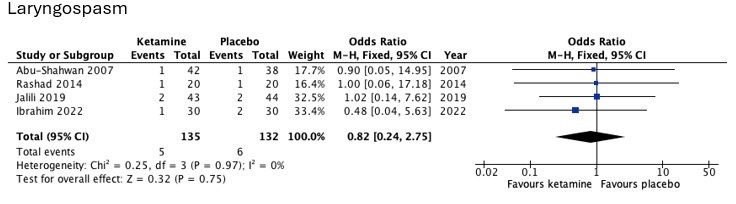

Supplement: Supplementary file 1 [file mmc1.docx]
